# Diversity and Physiological Characteristics of Antarctic Lichens-Associated Bacteria

**DOI:** 10.3390/microorganisms9030607

**Published:** 2021-03-15

**Authors:** Hyun-Ju Noh, Yerin Park, Soon Gyu Hong, Yung Mi Lee

**Affiliations:** 1 Division of Life Sciences, Korea Polar Research Institute, 26 Songdomirae-ro, Yeonsu-gu, Incheon 21990, Korea; nhjoo@kopri.re.kr (H.-J.N.); ellen55@kopri.re.kr (Y.P.); polypore@kopri.re.kr (S.G.H.); 2 School of Earth and Environmental Sciences, Seoul National University, Gwanak-gu, Seoul 08826, Korea

**Keywords:** Antarctic lichens, lichen-associated bacteria, macromolecule hydrolysis, indole-3-acetic acid, phosphate solubilization, nitrogen fixation

## Abstract

The diversity of lichen-associated bacteria from lichen taxa *Cetraria*, *Cladonia*, *Megaspora*, *Pseudephebe*, *Psoroma*, and *Sphaerophorus* was investigated by sequencing of 16S *rRNA* gene amplicons. Physiological characteristics of the cultured bacterial isolates were investigated to understand possible roles in the lichen ecosystem. Proteobacteria (with a relative abundance of 69.7–96.7%) were mostly represented by the order Rhodospirillales. The 117 retrieved isolates were grouped into 35 phylotypes of the phyla Actinobacteria (27), Bacteroidetes (6), Deinococcus-Thermus (1), and Proteobacteria (Alphaproteobacteria (53), Betaproteobacteria (18), and Gammaproteobacteria (12)). Hydrolysis of macromolecules such as skim milk, polymer, and (hypo)xanthine, solubilization of inorganic phosphate, production of phytohormone indole-3-acetic acid, and fixation of atmospheric nitrogen were observed in different taxa. The potential phototrophy of the strains of the genus *Polymorphobacter* which were cultivated from a lichen for the first time was revealed by the presence of genes involved in photosynthesis. Altogether, the physiological characteristics of diverse bacterial taxa from Antarctic lichens are considered to imply significant roles of lichen-associated bacteria to allow lichens to be tolerant or competitive in the harsh Antarctic environment.

## 1. Introduction

Lichens form symbiotic relationships between fungi (mycobiont) and photosynthetic algae and/or cyanobacteria (photobiont) [1,2,3]. Establishment of symbiotic lifestyles through the proper combinations of fungal and algal species is considered to support the ecological success of lichens as one of the earliest colonizers of severe habitats and recently exposed land. Furthermore, lichens are usually highly tolerant to harsh environmental conditions such as extremes in temperature, low water content, low nutrient availability, and high ultraviolet light intensities. These properties enable more than 19,000 lichen species to thrive in practically any terrestrial environment from polar areas to tropical rainforests, covering up to 8% of the total land surface [3,4,5,6].

Despite the general definition of lichens as bipartite or tripartite according to the mycobiont and photobiont symbioses, the presence of other microorganisms such as lichen-associated fungi, microalgae, and bacteria in the lichen thalli as an additional and integral component of lichen has long been revealed [7,8,9,10,11,12,13,14,15,16,17,18,19,20,21,22,23,24,25,26]. With the observation of high bacterial diversity and abundance in lichen, polyphasic approaches including metagenomic analysis and physiological determination of cultured isolates have been applied to understand the roles of bacterial communities in the lichen symbioses. As a consequence, several putative functional roles of bacteria such as nitrogen fixation, phosphate solubilization, amino acids and phytohormones production, nutrient supply via hydrolysis of major organic compounds, and defense mechanisms through antagonistic activities within the lichen symbiosis have been suggested, and these functions are considered to help lichens survive in nutrient-poor and unfavorable habitats with the partnership between mycobiont and photobiont [8,18,26,27,28,29,30,31,32,33,34].

Among the bacterial groups, the class Alphaproteobacteria dominated across different lichen species, accounting for up to 60–70% of bacterial communities in lichens across diverse geographical areas [15,21,26,28,35,36,37,38]. At the order level, Alphaproteobacteria was represented by Rhizobiales, Rhodospirillales, and Sphingomonadales at different proportions depending on the species or locality where the lichens were collected [11,15,21,25,29,37,38]. Despite their dominance, little is known about the functions of Alphaproteobacteria members in the lichen symbioses. Metagenomic analysis of *Lobaria pulmonaria* that focused on the functions of order Rhizobiales revealed metabolic pathways supporting the symbiosis, including auxin and vitamin production, nitrogen fixation, and stress protection [39]. However, a high portion of the metagenomic assignments could not be placed in any of the known Rhizobiales lineages, indicating the presence of numerous undescribed taxa in this lineage, which is consistent with the dominance of the undescribed lineage named lichen-associated Rhizobiales-1 (LAR1) across varying lichen samples [13]. Many isolates (112/234) belonging to the LAR1 lineage were successfully cultivated from *Umbilicaria esculenta* (Miyoshi), *Parmelia omphalodes* (L.) Ach., and *Lobaria retigera* (Bory) Trevis. [33]. Amplification of the *nifH* gene revealed that many isolates of the order Rhizobiales were *nifH* positive, demonstrating their role in nitrogen fixation. However, further physiological examination to verify these functions in the lichen symbioses has not been performed using those isolates. Similar to the order Rhizobiales, the order Rhodospirillales accounted for a high proportion of Alphaproteobacteria in some chlorolichens or Antarctic lichens [15,21,25,26]. Rhodospirillales in lichens was likely able to fix nitrogen because there are several known nitrogen-fixing bacteria in this lineage [40,41,42,43]. Recently, two bacterial isolates, *Lichenicola cladoniae* and *Lichenicoccus roseus* of the order Rhodospirillales from lichens *Cladonia borealis* S. Stenroos from Antarctica and *Cladonia arbuscula* (Wallr.) Rabenh. from Russia, respectively, have been reported [44,45]. Strains of the order Rhizobiales, *Lichenihabitans psoromatis* from a lichen *Psoroma* collected from Antarctica and *Lichenibacterium ramalinae* and *Lichenibacterium minor* from a lichen *Ramalina* collected from Russia, were isolated and physiological characteristics were determined [46,47]. These strains did not have the *nifH* gene, indicating that they are not capable of fixing atmospheric nitrogen. These previous results raise the necessity to obtain further isolates to validate and extend our knowledge about the role of common and dominant taxa in the lichen symbioses.

The aim of this study was to obtain bacterial isolates belonging to Alphaproteobacteria, the dominant lineage (accounting for 69.5–95.9% of the total bacteria) from six Antarctic lichen genera, *Cetraria*, *Cladonia*, *Megaspora*, *Pseudephebe*, *Psoroma*, and *Sphaerophorus*. To understand the functions of cultivated isolates in the lichen symbioses, physiological characteristics including extracellular enzymes secretion, hormone production, phosphate solubilization, and nitrogen fixation were investigated.

## 2. Materials and Methods

### 2.1. Sample Collection and Sampling Sites

Six samples of *Cetraria islandica* (L.) Ach., *Cladonia borealis* S. Stenroos, *Megaspora verrucosa* (Ach.) Hafellner & V. Wirth, *Pseudephebe pubescens* (L.) M. Choisy, *Psoroma cinnamomeum* Malme, and *Sphaerophorus globosus* (Huds.) Vain. were collected from Barton Peninsula, King George Island, Antarctica, from December 2011 to February 2012 (Table 1) and identified based on 28S *rRNA* gene sequences obtained by PacBio RSII platform (Pacific Bioscience, Menlo Park, CA, USA). The study area, the Barton Peninsula, is the southwestern part of King George Island, stretching 540 km off the northern end of the Antarctic Peninsula.

The Barton Peninsula has a rugged topography with a wide and gentle slope in the central belt, having altitudes ranging from 90 to 180 m above sea level [48]. Fine fractions of soils on the Barton Peninsula are mostly composed of mineral and rock fragments with some volcanic ashes [48]. The Barton Peninsula is a cold moist maritime Antarctic region, which is characterized by annual average temperature ranges of −1.8~1.6 °C, an annual average relative humidity of 89%, and an annual precipitation rate of 437.6 mm [48]. Sixty-eight lichen species and 32 moss species with two vascular plants of *Deschampsia antarctica* and *Colobanthus quitensis* have been reported in this area [49]. A chisel was used to obtain the samples, and they were transported to the laboratory in Korea at −20 °C, where they were preserved at −20 °C until use.

### 2.2. Genomic DNA Extraction, Amplification, and Bacterial Community Analysis

Samples were ground into fine powder using the TissueLyser II (Qiagen, Hilden, Germany) after freeze-drying, and genomic DNA was extracted from approximately 0.5 g of sample using an Exgene Soil DNA mini kit (GeneAll, Seoul, Korea) in accordance with the manufacturer’s instructions. The V4–V5 regions of the bacterial 16S *rRNA* genes were amplified with polymerase chain reaction using the primer pair 515F/926R [43,50] at Integrated Microbiome Resource (Dalhousie University, Halifax, N.S., Canada, http://cgeb-imr.ca accessed on 29 January 2021) in 2017. Sequencing of the amplicons was conducted by IMR using the paired-end (2 × 300 bp) Illumina MiSeq system (Illumina, San Diego, CA, USA). The adapter and primer sequences were removed using Cutadapt v2.10 [51], and the resultant sequences were processed by DADA2 v0.9.5 [52] to infer amplicon sequence variants (ASVs), which allowed a single nucleotide resolution. For quality trimming, a relaxed filtering option on the reverse reads as maxEE = c (2, 2) was applied, and the low-quality tails of each forward and reverse read were removed with truncLen = c (270, 210). Read pairs were then denoised based on a DADA2 error model, merged, and chimeras were removed by de novo approach. Representative ASV sequences were then taxonomically assigned against the EzBiocloud database [53]. All sequence data used in this study are deposited to the Sequence Read Archive (SRA) at NCBI (National Center for Biotechnology Information, Bethesda, MD, USA) under the accession numbers SRR13490317–SRR13490322.

### 2.3. Isolation of Bacterial Strains

The specimens were washed for 10 min in 1 mL of 0.85% NaCl by vortexing in a Multi-EP tube vortexer (FinePCR, Gyeonggi, Korea) followed by centrifugation at 10,000 rpm (Eppendorf, Hamburg, Germany) for 5 min, discarding the supernatant. The process was repeated four times. After the final wash, the samples were crushed in a TissueLyzer II containing steel beads (Qiagen, Hilden, Germany) twice for 2 min. One hundred microliters of the final suspension was then spread on 0.01 × Reasoner’s 2A (R2A), 0.1 × R2A, 0.01 × Inorganic-Salts Starch (ISP4), 0.1 × ISP4, Malt-Yeast extract (MY), and Nitrogen-fixing bacteria (Nfb) [54] solid media. The plates were incubated at 10 °C for 25 days. After the incubation, bacterial colonies from the agar plates were picked based on their morphology and subcultured on fresh R2A agar medium three or more times until pure isolates were obtained. Pure cultures of the bacterial isolates were preserved in 20% (*v*/*v*) glycerol at −80 °C.

### 2.4. Identification of Bacterial Isolates

Genomic DNA of cultured isolates was extracted by using the Mini Tissue DNA kit (Cosmo Genetech Inc., Seoul, Korea) in accordance with the manufacturer’s instructions. The 16S *rRNA* gene was amplified with two universal primers, 518F and 800R [55]. PCR products were purified using LaboPass PCR purification kit (Cosmo Genetech Inc., Seoul, Korea) and sequenced with the same primers used for amplification. The sequences of the 16S *rRNA* gene were compared with those of type strains available in the EzBioCloud database (ChunLab Inc., Seoul, Korea) [53] to find closely related species and to choose reference sequences for the phylogenetic analyses. Phylogenetic trees of the 16S *rRNA* gene sequences were reconstructed by the maximum likelihood method based on the GTR evolutionary model and the search options of best tree topology finding by branch swapping of NNIs and SPRs using MEGA X program [56]. The robustness of the tree topologies was assessed by bootstrap analyses based on 1000 replications of the sequences. The species affiliation of a bacterial isolate was determined when the isolate formed a monophyletic group with the reference species and the sequence similarity was 98.65% or higher. The sequences were deposited in GenBank (National Center for Biotechnology Information, Bethesda, MD, USA) under the accession numbers MW507603–MW507719.

### 2.5. Examination of Bacterial Physiological Characteristics

To determine physiological characteristics, cell suspensions were replica plated on square dishes of diverse solid media with a 96-pin replicator (VP-408B, V&P Scientific, San Diego, CA, USA). Cell suspensions were prepared by adding a half-full loop (5 mm diameter) of cells from the agar plates to 500 μL of 0.85% NaCl with vigorous shaking using a vortex mixer. To inoculate bacterial isolates, 200 μL of the suspension was then transferred to 96-well plates. For the determination of optimal growth temperature, cell suspensions were replica plated on R2A with a 96-pin replicator and then incubated for 21 days at 0, 4, 10, 15, 20, 25, 30, or 37 °C. Growth was evaluated by scoring the size and turbidity of the colonies as described in Lee et al. [56]. Secretion of extracellular protease, lipase, amylase, and chitinase was examined by replica plating of the cell suspensions onto 0.1 × R2A plates supplemented with 1% skim milk (Fluka, Dorset, UK), 1% tributyrate (Sigma-Aldrich, St. Louis, MO, USA), 1% starch (Alfa Aesar, Ward Hill, MA, USA), or 1% chitin (Wako Chemicals, Richmond, VA, USA), respectively, and incubating at 10 °C for 21 days. Enzyme activity was expressed as scores based on the ratio of colony size to the width of the clear zone surrounding the colony as described in Lee et al. [57]. Hydrolysis of polymer and purine utilization were examined on 0.1 × R2A plates supplemented with Tween 20 (Duksan, Gyeonggi, Korea), 40, 60, or 80 (Sigma-Aldrich) and xanthine (Avocado Research Chemicals Ltd., London, UK) or hypoxanthine (Sigma-Aldrich), respectively.

Mineral phosphate solubilization activity was assayed on Pi solid medium (glucose, 10.0 g; (NH4)_2_SO_4_, 0.5 g; yeast extract powder, 0.5 g; NaCl, 0.3 g; KCl, 0.3 g; FeSO_4_ 7H_2_O, 0.03 g; MgSO_4_ 7H_2_O, 0.3 g; MnSO_4_ 4H_2_O, 0.03 g; Ca_3_(PO4)_2_, 5.0 g; agar, 20.0 g; pH 7.0–7.5 in 1 L) [58,59]. The clearance zone around the colony was observed after 21 days incubation at 10 °C.

Indole-3-acetic acid (IAA) excretion by bacterial strains was determined by means of a modified colorimetric analysis [60]. Cells from the agar plates were inoculated into 1 mL of R2B medium with L-tryptophan (0.2 g/L) in triplicate. After cultivation at 15 °C and 150 rpm shaking in the absence of light until OD600 of the cell suspension was approximately 0.5, and cultures were centrifuged at 10,000 rpm for 10 min. The cell-free supernatant was mixed with Salkowski reagent (50.0 mM FeCl_3_, 35.0% (*v*/*v*) perchloric acid) at the ratio of 3:2 and incubated for 30 min in the absence of light. IAA concentration was measured spectrophotometrically using an Envision microplate reader (PerkinElmer, Waltham, MA, USA) at 530 nm; the IAA concentration was calculated according to a standard curve.

Nitrogen fixation ability was tested by cultivation on Nfb solid medium. Isolates growing with scores of ≥3 on the Nfb solid medium were denoted as nitrogen-fixing bacteria.

### 2.6. Genome Sequencing and Analysis

Genomic sequences of PAMC 29362 and PAMC 29367 of the genus *Polymorphobacter* were obtained by sequencing with Illumina HiSeq (Macrogen, Seoul, Korea) and assembled with Unicycler assembler version 0.4.9b [61]. Genome annotation was performed using the Rapid Annotation using Subsystems Technology (RAST) server [62]. Analysis of the Kyoto Encyclopedia of Genes and Genomes (KEGG) orthology was performed using KEGG Automatic Annotation Server (KAAS) (Kyoto University, Uji, Kyoto, Japan) [63]. The GenBank/EMBL/DDBJ accession numbers for the draft genome sequences of PAMC 29362 and PAMC 29367 are deposited under BioProject ID PRJNA693408.

## 3. Results and Discussion

### 3.1. Bacterial Community Structure Based on the Cultivation-Independent and -Dependent Approaches

Sequencing of the 16S *rRNA* genes in genomic DNA extracted from six lichens of the genera *Cetraria*, *Cladonia*, *Megaspora, Pseudephebe*, *Psoroma*, and *Sphaerophorus* produced 6501 to 14,292 reads after the quality check (Table S1). Plastid and non-bacterial sequences were excluded from further analyses. A total of 297 ASVs were observed, and each sample included from 23 to 112 ASVs (Table S1). Proteobacteria (69.7–96.7%) and Acidobacteria (0.3–30.2%) dominated across the samples (Figure S1). At the class level of Proteobacteria, Alphaproteobacteria accounted for most of the Proteobacteria, ranging from 69.5 to 95.9% (Figure S1). At the order level, Alphaproteobacteria was mostly represented by Rhodospirillales (64.2–87.7%), followed by Rhizobiales (0.2–13.0%), Caulobacterales (0.0–2.1%), and Sphingomonadales (0.0–0.5%) (Figure S1). Rhodospirillales accounted for a higher proportion than Rhizobiales across the six samples; the proportion of Rhizobiales was more than 10% of the bacterial communities only in lichens of *Megaspora* (12.1%) and *Psoroma* (13.0%) (Figure S1). Approximately 400 lichen species are known in Antarctica, and lichens are a major terrestrial vegetation type in Antarctica [64,65]. Lichen-associated bacteria are considered to contribute to the tolerance of lichens to the extreme environments of Antarctica. However, only a few studies on the bacterial communities of lichens from Antarctica based on culture-independent approaches have been performed to date [21,25,38]. This study provides the first report of the bacterial communities of *Megaspora*, *Pseudephebe, Psoroma,* and *Sphaerophorus* based on the cultivation-independent approach. Bacterial communities of lichens are structured by various factors such as host, photobiont, geography, and substrates [15,21,25,26,37,38]. Consistent with previous studies on the Antarctic lichen microbiome of *Amandinea*, *Buellia*, *Cetraria*, *Cladonia*, *Ochrolechia*, *Umbilicaria*, and *Usnea*, Alphaproteobacteria was the major bacterial class with higher proportions of Rhodospirillales than that of Rhizobiales at the order level [21,25,38].

**Figure 1:**
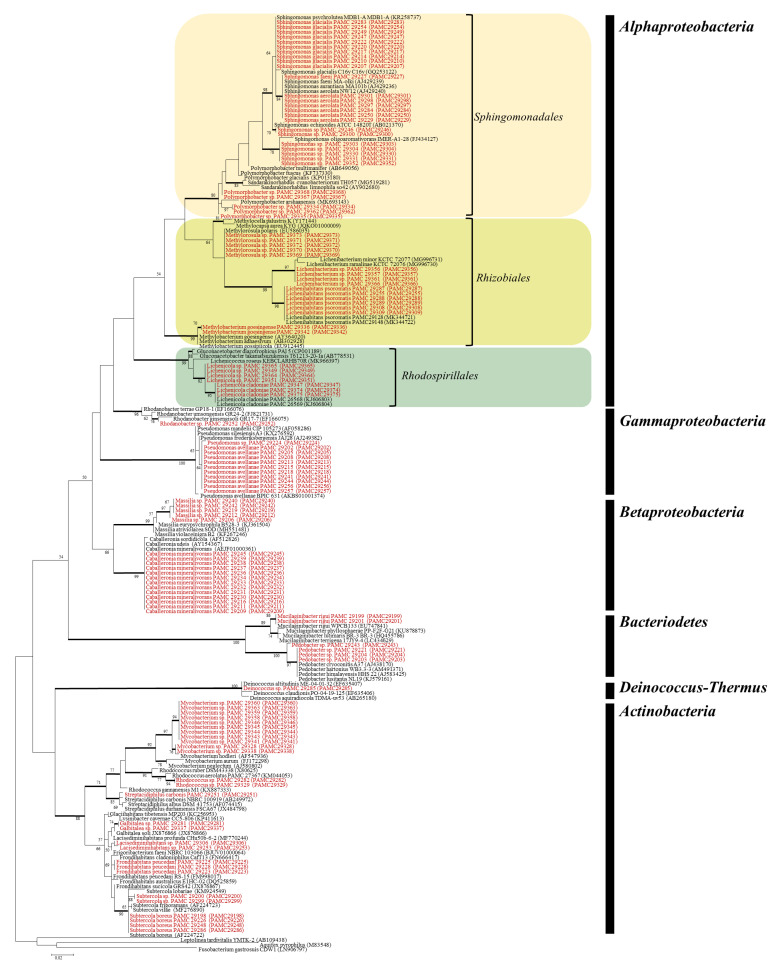
Maximum likelihood tree of isolates with closely related reference species based on 16S *rRNA* gene sequences. Representative isolates for each phylotype are indicated by bold letters. Branches supported by high bootstrap values (>70%) are shown as thick lines.

The bacterial 16S *rRNA* gene sequences of 117 isolates obtained by cultivation belonged to Alphaproteobacteria (53), Actinobacteria (27), Betaproteobacteria (18), Gammaproteobacteria (12), Bacteroidetes (6), and Deinococcus-Thermus (1) (Figure 1 and Table 2). Seven alphaproteobacterial genera were present, including *Lichenibacterium* (4), *Lichenihabitans* (6), *Methylobacterium* (2), *Methylorosula* (5) of the order Rhizobiales, *Lichenicola* (7) of the order Rhodospirillales, and *Polymorphobacter* (5) and *Sphingomonas* (24) of the order Sphingomonadales (Figure 1 and Table 2). Twenty-seven isolates of the phylum Actinobacteria were represented by the genera *Frondihabitans* (3), *Galbitalea* (2), *Lacisediminihabitans* (2), *Mycobacterium* (11), *Subtercola* (6), *Rhodococcus* (2), and *Streptacidiphilus* (1) (Table 2). Eighteen isolates of Betaproteobacteria were represented by the genera *Caballeronia* (13) and *Massilia* (5). Twelve isolates of Gammaproteobacteria were represented by the genera *Rhodanobacter* (1) and *Pseudomonas* (11). Bacteroidetes were represented by the genera *Mucilaginibacter* (2) and *Pedobacter* (4). Only one isolate of *Deinococcus* of Deinococcus-Thermus was cultivated in this study. The number of bacterial isolates from *Megaspora* specimen (51/117) was highest followed by *Psoroma* (39/117) and *Cladonia* (15/117). Among 34 phylotypes obtained in this study, 19 phylotypes were obtained in the lichen *Megaspora* and 12 phylotypes in *Psoroma* (Figure 1 and Table 2). Members of the genera *Frondihabitans*, *Galbitalea*, *Mycobacterium*, *Subtercola*, *Rhodococcus*, *Methylobacterium*, *Polymorphobacter*, *Mucilaginibacter*, and *Deinococcus* were cultivated only in *Megaspora* (Table 2). Members of the genera *Lichenibacterium* and *Lichenihabitans* previously known as LAR1 lineage were obtained from *Megaspora* and *Psoroma* whereas members of the order Rhodospirillales represented by genus *Lichenicola* were isolated from *Cladonia*, *Pseudephebe*, and *Sphaerophorus* (Figure 1 and Table 2). Comparing the phylogenetic position and cultured diversity with previous studies by Lee et al. and Jiang et al. [18,33], taxa belonging to the genera *Sphingomonas*, *Aureimonas*, *Methylobacterium*, *Caballeronia*, *Massilia*, and *Pseudomonas* were commonly obtained from lichens (Figure S2) indicating the possibility that they play a vital role for lichens regardless of the lichen species and localities. However, members of the genus *Polymorphobacter* were obtained only in this study, and members of *Rhodanobacter*, *Lichenicola*, *Lichenibacterium,* and *Lichenihabitans* were only isolated from Antarctic lichens (Figure S2).

The overall similarity of 16S *rRNA* gene sequences of the bacterial isolates to the known type strains ranged from 92.6% to 100%, and 46 isolates had ≤98.65% similarity, the threshold for differentiating two species [66], indicating that they are potential novel species. When comparing the cultivation-independent results with cultured isolates, 28 ASVs out of 50 major ASVs with more than 1% in relative abundance of bacterial communities in at least one sample showed low similarity (91.5–97.9%) with cultured isolates obtained in this study (Table S3). In particular, ASV00007, ASV00020, and ASV00027 of the order Rhodospirillales with proportions of >24% of bacterial communities in each sample showed 95.4–96.3% 16S *rRNA* gene similarity with PAMC 29349 (Table S3), indicating that traditional cultivation methods have failed to obtain ecologically more relevant microorganisms in this study. Nonetheless, lichen-associated isolates of the genus *Polymorphobacter* from crustose *Megaspora* cultured only in this study are very interesting. The strains of the genus *Polymorphobacter* growing at low temperatures (−4 °C to 4 °C) were inferred to be an aerobic anoxygenic photoheterotrophic bacterium, based on the presence of the photosynthetic gene *pufML* or bacteriochlorophyll a [67,68]. *Polymorphobacter* sp. strains in this study had 94.8–97.0% 16S *rRNA* gene sequence similarity with *Polymorphobacter arshaanensis* and genome sequence of PAMC 29362 and PAMC 29367 revealed that these strains include most of genes involved in photosynthesis and synthesis of bacteriochlorophyll a (Table S4). Phototrophy by lichen-associated bacteria that harvest sunlight abundantly present during the austral summer in Antarctica and convert it into chemical energy might enhance the survival of lichens in the oligotrophic environment [69]. Thus, despite the cultivation anomaly not fully reflecting the environmental diversity, cultured isolates provide the ability to test putative functions to fill the gap regarding the functional capacities inferred from molecular approaches.

### 3.2. Physiological Characteristics

**Figure 2:**
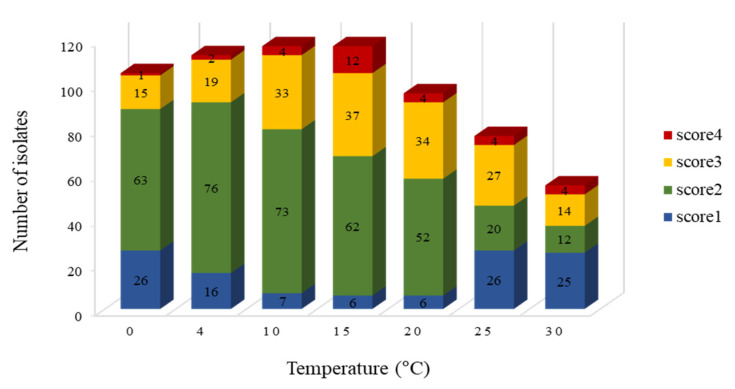
Effect of temperature on bacterial growth. The degree of growth was scored from 1 to 4, with a higher number indicating better growth.

All 117 bacterial isolates grew at 10 and 15 °C and most of the strains (105) were able to grow at 4 °C. However, the number of strains that can grow at higher temperature decreased to 96, 77, and 55 at 20, 25, and 30 °C, respectively. No strains could grow at 37 °C (Figure 2).

Extracellular protease activities were detected in 9 isolates affiliated with *Streptacidiphilus carbonis* (1), *Sphingomonas gracilis* (2), *Massilia* sp. (A) (1), *Mucilaginibacter rigui* (2), and *Pedobacter* sp. (B) (3), (Table 3 and Table S2). *Streptacidiphilus carbonis* PAMC 29251 isolated from lichen of *Sphaerophorus* showed high extracellular protease activity with a score of 4. The protease activity of other strains scored ≤3. None of the isolates exhibited lipase, amylase, or chitinase activities. Hydrolysis of polymers such as Tween 20 or Tween 60 was observed in 17 isolates of *Galbitalea* sp. (1), *Mycobacterium* sp. (A) and (B), *Rhodococcus* sp. (1), and *Caballeronia mineralivorans* (6) (Table 3). Hydrolysis of xanthine was observed in 14 isolates of the genera *Streptacidiphilus* (1) and *Caballeronia* (13), and hydrolysis of hypoxanthine was observed in 12 isolates of the genera *Subtercola* (1), *Streptacidiphilus* (1), *Sphingomonas* (3), and *Caballeronia* (7), supporting the putative involvement of lichen-associated bacteria in nutrient cycling in the lichens.

Thirty isolates of the phylotypes Subtercola boreus (1), Lichenihabitans psoromatis (2), Lichenicola cladoniae (2) and Lichenicola sp. (2), Caballeronia mineralivorans (10), Massilia sp. (A) (2), Rhodanobacter sp. (1), and Pseudomonas sp. (A) and (B) (10) showed the ability to solubilize phosphate (Table 3 and Table S2). High phosphate solubilization activity (≥3) was observed in the strains belonging to Lichenicola, Caballeronia, Massilia, and Pseudomonas (Table S2). Phosphorus is a primary element involved in all major metabolic pathways [70,71]. In nature, large portions of phosphorus are present in insoluble form that cannot be directly used by organisms [72]. Interestingly, isolates of Lichenicola (order Rhodospirillales) and Lichenihabitans psoromatis (order Rhizobiales) were capable of solubilizing inorganic phosphate. Although bacterial taxa belonging to orders Rhodospirillales and the LAR1 lineage of Rhizobiales dominated across diverse lichens, only a few bacterial taxa belonging to orders Rhodospirillales and the LAR1 lineage of Rhizobiales have been successfully cultivated from lichen [18,33,44,45,46,47]. The phosphate-solubilizing ability of those strains has not yet been determined [27]. Most strains (10/13) of the genus Caballeronia solubilized inorganic phosphate in this study. Considering that members of Caballeronia were commonly obtained from Arctic and Antarctic lichens [18] and identification of the capability of Caballeronia strains in this study to solubilize inorganic phosphate, phosphate solubilizing lichen-associated bacteria could be of great importance for lichen colonization in the oligotrophic environments of Antarctica.

IAA was detected in the supernatant of 67 bacterial culture (57%) (Table 3). Secretion of IAA (more than 5 ng/μL) was detected in isolates belonging to the genera *Frondihabitans*, *Galbialea*, *Lacisediminihabitans*, *Subtercola*, *Rhodococcus*, *Lichenibacterium*, *Lichenihabitans*, *Methylobacterium*, *Lichenicola*, *Polymorphobacter*, *Sphingomonas*, *Caballeronia*, *Massilia*, *Rhodanobacter*, *Pseudomonas*, and *Pedobacter*. Production of IAA has been reported in lichen, lichen-forming fungi and lichen-associated bacteria [8,29,73,74,75]. IAA from lichen-forming fungi enhances the water uptake in higher plants and is also related to promotion of cell growth, biomass production, and intracellular concentrations of chlorophyll a, carotenoids, and lipids biosynthesis of the green algae *Scenedesmus quadricauda* [76,77]. Pichler et al. showed that three lichen forming fungi—*Cladonia grayi* Sandst., *Xanthoria parietina* (L.) Th. Fr., and *Tephromela atra* (Huds.) Hafellner—produced IAA that was secreted exogenously and increased the water contents of their microalgae by up to 4.4% [74]. Thus, it was suspected that bacterial IAA in lichen supports the amicable interaction between lichen-associated bacteria and the microalgae as well as the lichen forming fungi in lichen symbiosis [12,29]. This could dramatically help the symbiotic relationship between algae with lichen forming fungi when it comes to a certain environmental status that can resume photosynthesis and thus support lichen survival.

Thirty-five strains of the phylotypes *Frondihabitans peucedani* (3), *Lacisediminihabitans* sp. (A), *Mycobacterium* sp. (A) and (B) (9), *Lacisediminihabitans* sp. (A) (1), *Subtercola boreus* (1), *Methylorosula* sp. (2), *Sphingomonas aerolata* (3), *Sphingomonas gracilis* (1), *Caballeronia mineralivorans* (13), and *Pedobacter* sp. (A) and (B) (2) grew on Nfb medium (Table 3). All cultured strains of *Caballeronia* in this study and *Methylorosula* sp., *Sphingomonas aerolata*, and *S. glacialis* grew on Nfb medium. The ability of members of *Caballeronia* (previously known as Burkholderia) to fix atmospheric nitrogen is known [78] and the nifH gene, responsible for production of the nitrogenase Fe protein subunit, was successfully amplified in some cultured isolates of this genus from lichen *Lobaria retigera* [33]. Because acetylene reduction assay-positive but nifH-negative isolates were reported [8,79], we cannot exclude the possibility that nifH-negative isolates can fix nitrogen via an alternative system or have divergent genes. Furthermore, 35% of the total isolates grown on Nfb medium in this study, may grow using the nitrogenous elements in agar instead of atmospheric nitrogen fixation. Order Rhodospirillales and the LAR1 lineage of Rhizobiales (Alphaproteobacteria) include many nitrogen-fixing taxa that can form plant-associated root nodules [40,41,42,43,80,81]. Previous studies based on molecular approaches suggested the nitrogen fixing function of these bacteria in lichen symbioses [12,27,39]. Interestingly, most of the bacterial isolates of orders Rhodospirillales and the LAR1 lineage obtained in this study did not grow on Nfb medium. Instead, many strains of the genus Sphingomonas of Sphingomonadales could fix nitrogen in this study. Nitrogen-fixing gammaproteobacterial isolates have been cultivated from cyanobacteria-deprived lichens [8]. However, gammaproteobacterial isolates in this study did not grow on Nfb medium. Both cyanolichens, which have symbiotic relationships with cyanobacteria to fix nitrogen in the cephalodium, and many chlorolichens can grow in nutrient poor environments [3,82]. Lichens harboring nitrogen fixers of diverse taxa of Actinobacteria, Alphaproteobacteria, Betaproteobacteria, and Bacteroidetes, which were detected across the lichens, could be competitive to survive in nitrogen-limiting environments.

In this study, the comparison between culture-dependent and culture-independent bacterial communities revealed a limited overlap. However, psychrotolerant bacterial isolates of diverse phylotypes showed lytic, nitrogen-fixing, and phosphate-solubilizing ability that could significantly enhance the mobilization of nutrients in lichen symbioses. In particular, strains of the orders Rhodospirillales and the LAR1 lineage of Rhizobiales, which were predominant in lichen-associated bacterial communities, did not have nitrogen-fixing ability. Instead, they solubilized inorganic phosphates, showing the importance of cultivation for testing the metabolic potential and expanding knowledge about the functions of dominant bacterial taxa in lichens. Furthermore, the phototrophic potential of lichen-associated bacteria suggested for the first time in this study will contribute to the significant roles of lichen-associated bacteria for lichens to adapt to the oligotrophic environment. Although the knowledge gap about bacterial functions in the context of lichen symbioses still exists, the bacterial isolates obtained in this study can be used to verify and better understand the functions of lichen-associated bacteria.

## Figures and Tables

**Table 1:** Information on the lichen specimens used in this study.

Scheme	Genus	Sample ID	Growth Type	Photobiont	Latitude	Longitude
2012KG-B0531	*Cetraria*	Ct	Fruticose	Chlorolichen	62, 14′10.334″ S	58, 43′08.263″ W
2012KG-B0306	*Cladonia*	Cl	Fruticose	Chlorolichen	62, 14′26.606″ S	58, 43′34.412″ W
2012KG-B0468-FD42	*Megaspora*	Ms	Crustose	Chlorolichen	62, 14′20.781″ S	58, 45′28.821″ W
2012KG-B0625-FD85	*Pseudephebe*	Pp	Fruticose	Chlorolichen	62, 13′46.984″ S	58, 46′52.696″ W
2012KG-B0524B0-FD16	*Psoroma*	Ps	Squamulose	Tripartite	62, 14′10.334″ S	58, 43′08.263″ W
2012KG-B0103-FD77	*Sphaerophorus*	Sp	Fruticose	Chlorolichen	62, 13′39.591″ S	58, 45′33.939″ W

**Table 2:** Distribution of bacterial isolates according to lichen.

Taxonomic Assignment			Isolated From *						Total Number of Isolates
Phylum or Class	Order	Phylotype	Ct	Cl	Ms	Pp	Ps	Sp	
Actinobacteria	Micrococcales	*Frondihabitans peucedani*	–	–	3	–	–	–	3
		*Galbitalea* sp.	–	–	2	–	–	–	2
		*Lacisediminihabitans* sp. (A)	–	–	–	–	1	–	1
		*Lacisediminihabitans* sp. (B)	–	–	–	–	1	–	1
		*Mycobacterium* sp. (A)	–	–	2	–	–	–	2
		*Mycobacterium* sp. (B)	–	–	9	–	–	–	9
		*Subtercola boreus*	–	–	4	–	–	–	4
		*Subtercola* sp.	–	–	2	–	–	–	2
	Mycobacteriales	*Rhodococcus* sp.	–	–	2	–	–	–	2
	Streptomycetales	*Streptacidiphilus carbonis*	–	–	–	–	–	1	1
Alphaproteobacteria	Rhizobiales	*Lichenibacterium* sp.	–	–	3	–	1	–	4
		*Lichenihabitans psoromatis*	–	–	–	–	6	–	6
		*Methylobacterium goesingense*	–	–	2	–	–	–	2
		*Methylorosula* sp.	2	3	–	–	–	–	5
	Rhodospirillales	*Lichenicola cladoniae*	–	2	–	1	–	–	3
		*Lichenicola* sp.	–	–	–	1	–	3	4
	Sphingomonadales	*Polymorphobacter* sp. (A)	–	–	2	–	–	–	2
		*Polymorphobacter* sp. (B)	–	–	2	–	–	–	2
		*Polymorphobacter* sp. (C)			1				1
		*Sphingomonas aerolata*	–	–	6	–	–	–	6
		*Sphingomonas faeni*	–	–	1	–	–	–	1
		*Sphingomonas glacialis*	–	–	4	–	6	–	10
		*Sphingomonas* sp. (A)	–	–	2	–	–	–	2
		*Sphingomonas* sp. (B)	1	3	–	–	1	–	5
Betaproteobacteria	Burkholderiales	*Caballeronia mineralivorans*	2	7	–	–	4	–	13
		*Massilia* sp. (A)	–	–	–	–	4	–	4
		*Massilia* sp. (B)	–	–	–	–	1	–	1
Gammaproteobacteria	Lysobacterales	*Rhodanobacter sp.*	–	–	–	–	–	1	1
	Pseudomonadales	*Pseudomonas* sp. (A)	–	–	1	–	–	–	1
		*Pseudomonas* sp. (B)	–	–	–	–	10	–	10
Bacteroidetes	Sphingobacteriales	*Mucilaginibacter rigui*	–	–	2	–	–	–	2
		*Pedobacter* sp. (A)	–	–	–	–	1	–	1
		*Pedobacter* sp. (B)	–	–	–	–	3	–	3
Deinococcus-Thermus	Deinococcales	*Deinococcus* sp.	–	–	1	–	–	–	1
Total			5	15	51	2	39	5	117

* Sample ID is indicated in Table 1.

**Table 3:** Physiological characteristics.

Phylum or Class (Order)	Phylotype	Number of Isolates ^a^	Physiological Characteristics ^b^						
			Protease	Polymer	Xanthine	Hypoxanthine	Phosphate Solubilization	Growth Hormone ^d^	Nitrogen Fixation ^e^
***Actinobacteria***	***Frondihabitans peucedani***	3	–	–	–	–	–	3	3
	*Galbitalea* sp.	2	–	1	–	–	–	2	–
	*Lacisediminihabitans* sp. (A)	1	–	–	–	–	–	1	1
	*Lacisediminihabitans* sp. (B)	1	–	–	–	–	–	1	–
	*Mycobacterium* sp. (A)	2	–	2	–	–	–	–	1
	*Mycobacterium* sp. (B)	9	–	5	–	–	–	–	8
	*Subtercola boreus*	4	–	–	–	1	1	1	1
	*Subtercola* sp.	2	–	–	–	–	–	–	–
	*Rhodococcus* sp.	2	–	1	–	–	–	2	–
	*Streptacidiphilus carbonis*	1	1	–	1	1	–	–	–
*Alphaproteobacteria*	*Lichenibacterium* sp.	4	–	–	–	–	–	1	–
(*Rhizobiales*)	*Lichenihabitans psoromatis*	6	–	–	–	–	2	1	–
	*Methylobacterium goesingense*	2	–	–	–	–	–	2	–
	*Methylorosula* sp.	5	–	–	–	–	–	–	2
*Alphaproteobacteria*	*Lichenicola cladoniae*	3	–	–	–	–	2	3	–
(*Rhodospirillales*)	*Lichenicola* sp.	4	–	–	–	–	2	1	–
*Alphaproteobacteria*	*Polymorphobacter* sp. (A)	2	–	–	–	–	–	–	–
(*Sphingomonadales*)	*Polymorphobacter* sp. (B)	2	–	–	–	–	–	2	–
	*Polymorphobacter* sp. (C)	1	–	–	–	–	–	1	–
	*Sphingomonas aerolata*	6	–	–	–	1	–	6	3
	*Sphingomonas faeni*	1	–	–	–	–	–	1	–
	*Sphingomonas glacialis*	10	2	–	–	1	–	10	1
	*Sphingomonas* sp. (A)	2	–	–	–	1	–	2	–
	*Sphingomonas* sp. (B)	5	–	–	–	–	–	3	–
*Betaproteobacteria*	*Caballeronia mineralivorans*	13	–	6 ^c^	13	7	10	4	13
	*Massilia* sp. (A)	4	1	–	–	–	2	4	–
	*Massilia* sp. (B)	1	–	–	–	–	–	1	–
*Gammaproteobacteria*	*Rhodanobacter sp.*	1	–	–	–	–	1	1	–
	*Pseudomonas* sp. (A)	1	–	–	–	–	1	1	–
	*Pseudomonas* sp. (B)	10	–	–	–	–	9	10	–
*Bacteroidetes*	*Mucilaginibacter rigui*	2	2	–	–	–	–	2	–
	*Pedobacter* sp. (A)	1	–	–	–	–	–	1	1
	*Pedobacter* sp. (B)	3	3	1	–	–	–	–	3
*Deinococcus-Thermus*	*Deinococcus* sp.	1	–	–	–	–	–	–	–

^a^ Numbers indicate the total number of cultured isolates of each phylotype. ^b^ Numbers indicate the number of isolates showing the positive activity. ^c^ Number indicates positive isolates for the hydrolysis of Tween 60 except that of *Caballeronia mineralivorans*, which indicates the result of hydrolysis of Tween 20. ^d^ Growth hormone indicates the production of phytohormone indole-3-acetic acid. ^e^ Nitrogen fixation was determined by growing on nitrogen-free medium. Isolates growing with scores (≥3) on the nitrogen-fixing bacteria (Nfb) solid medium were denoted as nitrogen-fixing bacteria.

## Data Availability

All sequence data used in this study are deposited to the Sequence Read Archive (SRA) at NCBI under the ac-cession numbers SRR13490317–SRR13490322. The sequences were deposited in GenBank under the accession numbers MW507603–MW507719. The GenBank/EMBL/DDBJ accession numbers for the draft genome sequences of PAMC 29362 and PAMC 29367 are deposited under BioProject ID PRJNA693408.

## Supplementary Materials

The following are available online at https://www.mdpi.com/2076-2607/9/3/607/s1. Figure S1: Bacterial compositions of 6 lichen samples at the phylum (A), class (B), order (C), and family (D) levels. Figure S2: Maximum likelihood tree of isolates with reference species in Lee et al. and Jiang et al., based on 16S rRNA gene sequences [18,33], Table S1: Summary of sequencing results. Table S2: Taxonomic assignments, physiology of the bacterial isolates, and their origin. Table S3: Major ASVs within 6 lichen samples and their closest isolates. Table S4: Genes involved photosynthesis and bacteriochlorophyll.
