# Supplementary material for: Diversity and Physiological Characteristics of Antarctic Lichens-Associated Bacteria
Source: Microorganisms. 2021 Mar 15;9(3):607. doi: 10.3390/microorganisms9030607 (PMC8001610; doi:10.3390/microorganisms9030607)
Supplement: Supplementary file 1 [file microorganisms-09-00607-s001.zip › lichen_bacteria_cultivation_figures_supplementary.pptx]

## Slide 1
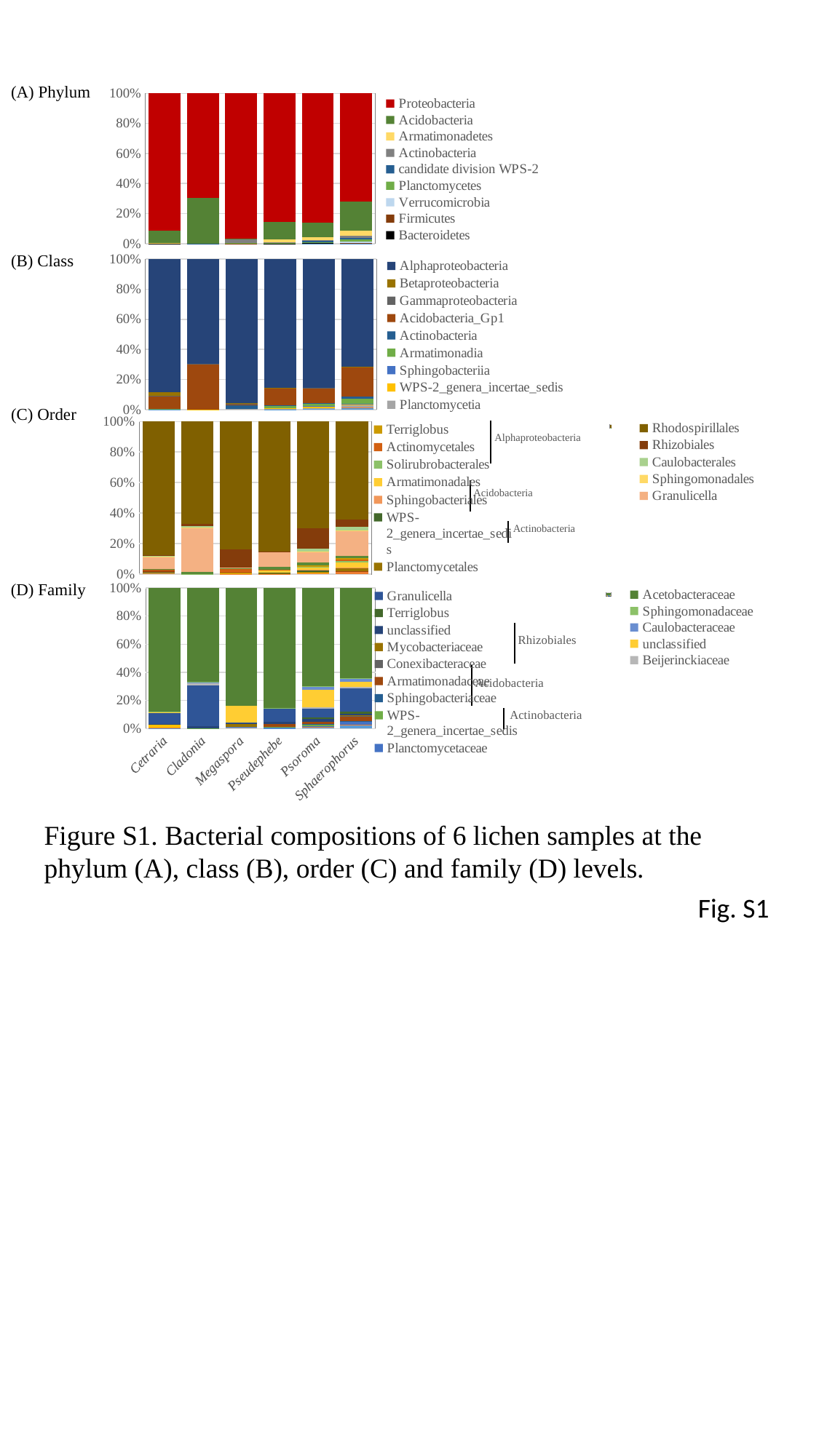

(A) Phylum
### Chart
| Category | candidate division WPS-1 | Cyanobacteria | Bacteroidetes | Firmicutes | Verrucomicrobia | Planctomycetes | candidate division WPS-2 | Actinobacteria | Armatimonadetes | Acidobacteria | Proteobacteria |
|---|---|---|---|---|---|---|---|---|---|---|---|
| Cetraria | 0.0 | 0.0 | 0.014964459408903853 | 0.17209128320239434 | 0.0 | 0.0 | 0.044893378226711564 | 0.014964459408903853 | 0.029928918817807706 | 8.477366255144034 | 91.24579124579122 |
| Cladonia | 0.0 | 0.0 | 0.0 | 0.0 | 0.0 | 0.013993842709207949 | 0.020990764063811923 | 0.013993842709207949 | 0.0 | 30.22670025188917 | 69.72432129862861 |
| Megaspora | 0.0 | 0.06152899553914783 | 0.0 | 0.030764497769573913 | 0.0 | 0.1076757421935087 | 0.0 | 2.861098292570374 | 0.0 | 0.2768804799261652 | 96.66205199200122 |
| Pseudephebe | 0.0 | 0.0 | 0.03690581635665781 | 0.12547977561263657 | 0.0 | 0.059049306170652495 | 0.6716858576911722 | 0.05166814289932093 | 1.8674343076468851 | 11.62533215234721 | 85.56244464127549 |
| Psoroma | 0.07779074290159471 | 0.07779074290159471 | 0.22040710488785167 | 0.0 | 0.47970958122650065 | 0.1037209905354596 | 1.4002333722287048 | 0.14261636198625696 | 1.5947102294826916 | 9.736807986516268 | 86.16621288733306 |
| Sphaerophorus | 0.11156563778356265 | 0.10412792859799182 | 0.10412792859799183 | 0.2528821123094087 | 0.5429527705466716 | 1.7329862402380067 | 0.714020081814801 | 1.5098549646708814 | 3.3990330978058756 | 19.390107846783195 | 72.13834139085162 |
### Chart
| Category | Others | Spartobacteria | Planctomycetia | WPS-2_genera_incertae_sedis | Sphingobacteriia | Armatimonadia | Actinobacteria | Acidobacteria_Gp1 | Gammaproteobacteria | Betaproteobacteria | Alphaproteobacteria |
|---|---|---|---|---|---|---|---|---|---|---|---|
| Cetraria | 0.17209128320239434 | 0.0 | 0.0 | 0.044893378226711564 | 0.014964459408903853 | 0.029928918817807706 | 0.014964459408903853 | 8.477366255144034 | 0.5387205387205387 | 2.109988776655443 | 88.59708193041527 |
| Cladonia | 0.0 | 0.0 | 0.013993842709207949 | 0.020990764063811923 | 0.0 | 0.0 | 0.013993842709207949 | 30.22670025188917 | 0.18191995521970333 | 0.0 | 69.54240134340891 |
| Megaspora | 0.2153514843870174 | 0.0 | 0.1076757421935087 | 0.0 | 0.0 | 0.0 | 2.861098292570374 | 0.2768804799261652 | 0.15382248884786956 | 0.5076142131979695 | 95.8775572988771 |
| Pseudephebe | 0.18452908178328906 | 0.0 | 0.059049306170652495 | 0.6716858576911722 | 0.0 | 1.8674343076468851 | 0.05166814289932093 | 11.603188662533215 | 0.08857395925597875 | 0.14024210215529967 | 85.33362857986421 |
| Psoroma | 0.24633735252171657 | 0.47970958122650065 | 0.1037209905354596 | 1.4002333722287048 | 0.22040710488785167 | 1.5947102294826916 | 0.14261636198625696 | 9.671982367431607 | 0.4667444574095682 | 0.0 | 85.67353818228962 |
| Sphaerophorus | 0.714020081814801 | 0.5429527705466716 | 1.7329862402380067 | 0.714020081814801 | 0.10412792859799183 | 3.3990330978058756 | 1.5098549646708814 | 19.390107846783195 | 0.1710673112681294 | 0.1859427296392711 | 71.53588694682037 |(B) Class
(C) Order
### Chart
| Category | Others | Pseudomonadales | Xanthomonadales | Burkholderiales | Planctomycetales | WPS-2_genera_incertae_sedis | Sphingobacteriales | Armatimonadales | Solirubrobacterales | Actinomycetales | Terriglobus | unclassified | Granulicella | Sphingomonadales | Caulobacterales | Rhizobiales | Rhodospirillales |
|---|---|---|---|---|---|---|---|---|---|---|---|---|---|---|---|---|---|
| Cetraria | 0.38159371492704824 | 0.0 | 0.44893378226711567 | 2.109988776655443 | 0.0 | 0.044893378226711564 | 0.014964459408903853 | 0.029928918817807706 | 0.0 | 0.014964459408903853 | 0.0 | 0.21698466142910586 | 8.140665918443698 | 0.07482229704451926 | 0.5387205387205387 | 0.29928918817807704 | 87.68424990647215 |
| Cladonia | 0.15393226980128744 | 0.0 | 0.027987685418415897 | 0.0 | 0.013993842709207949 | 0.020990764063811923 | 0.0 | 0.0 | 0.013993842709207949 | 0.0 | 0.0 | 1.413378113630003 | 28.813322138259164 | 0.2938706968933669 | 0.6437167646235658 | 1.686258046459558 | 66.9185558354324 |
| Megaspora | 0.2153514843870174 | 0.15382248884786956 | 0.0 | 0.5076142131979695 | 0.1076757421935087 | 0.0 | 0.0 | 0.0 | 0.0 | 2.861098292570374 | 0.0 | 0.1692047377326565 | 0.1076757421935087 | 0.0 | 0.0 | 12.090447623442547 | 83.78710967543454 |
| Pseudephebe | 0.27310304103926775 | 0.0 | 0.0 | 0.14024210215529967 | 0.059049306170652495 | 0.6716858576911722 | 0.0 | 1.8674343076468851 | 0.0 | 0.05166814289932093 | 0.0 | 1.9855329199881901 | 9.617655742545026 | 0.029524653085326247 | 0.08857395925597875 | 0.14762326542663123 | 85.06790670209628 |
| Psoroma | 0.9594191624530013 | 0.0259302476338649 | 0.3370932192402437 | 0.0 | 0.1037209905354596 | 1.4002333722287048 | 0.22040710488785167 | 1.5947102294826916 | 0.14261636198625696 | 0.0 | 0.7908725528328796 | 2.178140801244652 | 6.651108518086347 | 0.4667444574095683 | 2.0873849345261246 | 13.016984312200181 | 70.02463373525218 |
| Sphaerophorus | 1.547043510598736 | 0.0 | 0.1710673112681294 | 0.1859427296392711 | 1.7329862402380067 | 0.714020081814801 | 0.10412792859799183 | 3.3990330978058756 | 0.5503904797322424 | 0.9445890665674973 | 1.2867236891037561 | 1.4057270360728895 | 16.444775009297143 | 0.40163629602082557 | 1.9412420974339901 | 5.005578281889178 | 64.16511714391969 |
### Chart
| Category | Others | Pseudomonadales | Xanthomonadales | Burkholderiales | Planctomycetales | WPS-2_genera_incertae_sedis | Sphingobacteriales | Armatimonadales | Solirubrobacterales | Actinomycetales | Terriglobus | unclassified | Granulicella | Sphingomonadales | Caulobacterales | Rhizobiales | Rhodospirillales |
|---|---|---|---|---|---|---|---|---|---|---|---|---|---|---|---|---|---|
| Aspicilia | 0.2153514843870174 | 0.15382248884786956 | 0.0 | 0.5076142131979695 | 0.1076757421935087 | 0.0 | 0.0 | 0.0 | 0.0 | 2.861098292570374 | 0.0 | 0.1692047377326565 | 0.1076757421935087 | 0.0 | 0.0 | 12.090447623442547 | 83.78710967543454 |
| Cetraria | 0.38159371492704824 | 0.0 | 0.44893378226711567 | 2.109988776655443 | 0.0 | 0.044893378226711564 | 0.014964459408903853 | 0.029928918817807706 | 0.0 | 0.014964459408903853 | 0.0 | 0.21698466142910586 | 8.140665918443698 | 0.07482229704451926 | 0.5387205387205387 | 0.29928918817807704 | 87.68424990647215 |
| Cladonia | 0.15393226980128744 | 0.0 | 0.027987685418415897 | 0.0 | 0.013993842709207949 | 0.020990764063811923 | 0.0 | 0.0 | 0.013993842709207949 | 0.0 | 0.0 | 1.413378113630003 | 28.813322138259164 | 0.2938706968933669 | 0.6437167646235658 | 1.686258046459558 | 66.9185558354324 |
| Pseudephebe | 0.27310304103926775 | 0.0 | 0.0 | 0.14024210215529967 | 0.059049306170652495 | 0.6716858576911722 | 0.0 | 1.8674343076468851 | 0.0 | 0.05166814289932093 | 0.0 | 1.9855329199881901 | 9.617655742545026 | 0.029524653085326247 | 0.08857395925597875 | 0.14762326542663123 | 85.06790670209628 |
| Psoroma | 0.9594191624530013 | 0.0259302476338649 | 0.3370932192402437 | 0.0 | 0.1037209905354596 | 1.4002333722287048 | 0.22040710488785167 | 1.5947102294826916 | 0.14261636198625696 | 0.0 | 0.7908725528328796 | 2.178140801244652 | 6.651108518086347 | 0.4667444574095683 | 2.0873849345261246 | 13.016984312200181 | 70.02463373525218 |
| Sphaerophorus | 1.547043510598736 | 0.0 | 0.1710673112681294 | 0.1859427296392711 | 1.7329862402380067 | 0.714020081814801 | 0.10412792859799183 | 3.3990330978058756 | 0.5503904797322424 | 0.9445890665674973 | 1.2867236891037561 | 1.4057270360728895 | 16.444775009297143 | 0.40163629602082557 | 1.9412420974339901 | 5.005578281889178 | 64.16511714391969 |Alphaproteobacteria
Acidobacteria
Actinobacteria
(D) Family
### Chart
| Category | Others | Xanthomonadaceae | Pseudomonadaceae | Burkholderiaceae | Planctomycetaceae | WPS-2_genera_incertae_sedis | Sphingobacteriaceae | Armatimonadaceae | Conexibacteraceae | Mycobacteriaceae | unclassified | Terriglobus | Granulicella | Methylobacteriaceae | Beijerinckiaceae | unclassified | Caulobacteraceae | Sphingomonadaceae | Acetobacteraceae |
|---|---|---|---|---|---|---|---|---|---|---|---|---|---|---|---|---|---|---|---|
| Cetraria | 0.4339693228582117 | 0.4264870931537599 | 0.0 | 2.109988776655443 | 0.0 | 0.044893378226711564 | 0.0 | 0.029928918817807706 | 0.0 | 0.0 | 0.21698466142910586 | 0.0 | 8.140665918443698 | 0.029928918817807706 | 0.13468013468013468 | 0.13468013468013468 | 0.5387205387205387 | 0.07482229704451926 | 87.68424990647215 |
| Cladonia | 0.18191995521970333 | 0.0 | 0.0 | 0.0 | 0.013993842709207949 | 0.020990764063811923 | 0.0 | 0.0 | 0.013993842709207949 | 0.0 | 1.413378113630003 | 0.0 | 28.813322138259164 | 0.0 | 1.686258046459558 | 0.0 | 0.6437167646235658 | 0.2938706968933669 | 66.9185558354324 |
| Megaspora | 0.7229656975849869 | 0.0 | 0.15382248884786956 | 0.0 | 0.1076757421935087 | 0.0 | 0.0 | 0.0 | 0.0 | 2.861098292570374 | 0.1692047377326565 | 0.0 | 0.1076757421935087 | 0.0 | 0.0 | 12.090447623442547 | 0.0 | 0.0 | 83.78710967543454 |
| Pseudephebe | 0.4281074697372306 | 0.0 | 0.0 | 0.03690581635665781 | 0.059049306170652495 | 0.6716858576911722 | 0.0 | 1.8674343076468851 | 0.0 | 0.0 | 1.9855329199881901 | 0.0 | 9.617655742545026 | 0.022143489813994686 | 0.12547977561263654 | 0.0 | 0.08857395925597875 | 0.029524653085326247 | 85.06790670209628 |
| Psoroma | 1.1279657720731233 | 0.3370932192402437 | 0.0 | 0.0 | 0.1037209905354596 | 1.4002333722287048 | 0.22040710488785167 | 1.5947102294826916 | 0.0 | 0.0 | 2.178140801244652 | 0.7908725528328796 | 6.651108518086347 | 0.0 | 0.9853494100868662 | 12.031634902113314 | 2.0873849345261246 | 0.4667444574095683 | 70.02463373525218 |
| Sphaerophorus | 2.722201561918928 | 0.1190033469691335 | 0.0 | 0.0 | 1.7329862402380067 | 0.714020081814801 | 0.03718854592785422 | 3.3990330978058756 | 0.5503904797322424 | 0.07437709185570844 | 1.4057270360728895 | 1.2867236891037561 | 16.444775009297143 | 0.0 | 1.2569728523614727 | 3.7486054295277054 | 1.9412420974339901 | 0.40163629602082557 | 64.16511714391969 |
### Chart
| Category | Others | Xanthomonadaceae | Pseudomonadaceae | Burkholderiaceae | Planctomycetaceae | WPS-2_genera_incertae_sedis | Sphingobacteriaceae | Armatimonadaceae | Conexibacteraceae | Mycobacteriaceae | unclassified | Terriglobus | Granulicella | Methylobacteriaceae | Beijerinckiaceae | unclassified | Caulobacteraceae | Sphingomonadaceae | Acetobacteraceae |
|---|---|---|---|---|---|---|---|---|---|---|---|---|---|---|---|---|---|---|---|
| Aspicilia | 0.7229656975849869 | 0.0 | 0.15382248884786956 | 0.0 | 0.1076757421935087 | 0.0 | 0.0 | 0.0 | 0.0 | 2.861098292570374 | 0.1692047377326565 | 0.0 | 0.1076757421935087 | 0.0 | 0.0 | 12.090447623442547 | 0.0 | 0.0 | 83.78710967543454 |
| Cetraria | 0.4339693228582117 | 0.4264870931537599 | 0.0 | 2.109988776655443 | 0.0 | 0.044893378226711564 | 0.0 | 0.029928918817807706 | 0.0 | 0.0 | 0.21698466142910586 | 0.0 | 8.140665918443698 | 0.029928918817807706 | 0.13468013468013468 | 0.13468013468013468 | 0.5387205387205387 | 0.07482229704451926 | 87.68424990647215 |
| Cladonia | 0.18191995521970333 | 0.0 | 0.0 | 0.0 | 0.013993842709207949 | 0.020990764063811923 | 0.0 | 0.0 | 0.013993842709207949 | 0.0 | 1.413378113630003 | 0.0 | 28.813322138259164 | 0.0 | 1.686258046459558 | 0.0 | 0.6437167646235658 | 0.2938706968933669 | 66.9185558354324 |
| Pseudephebe | 0.4281074697372306 | 0.0 | 0.0 | 0.03690581635665781 | 0.059049306170652495 | 0.6716858576911722 | 0.0 | 1.8674343076468851 | 0.0 | 0.0 | 1.9855329199881901 | 0.0 | 9.617655742545026 | 0.022143489813994686 | 0.12547977561263654 | 0.0 | 0.08857395925597875 | 0.029524653085326247 | 85.06790670209628 |
| Psoroma | 1.1279657720731233 | 0.3370932192402437 | 0.0 | 0.0 | 0.1037209905354596 | 1.4002333722287048 | 0.22040710488785167 | 1.5947102294826916 | 0.0 | 0.0 | 2.178140801244652 | 0.7908725528328796 | 6.651108518086347 | 0.0 | 0.9853494100868662 | 12.031634902113314 | 2.0873849345261246 | 0.4667444574095683 | 70.02463373525218 |
| Sphaerophorus | 2.722201561918928 | 0.1190033469691335 | 0.0 | 0.0 | 1.7329862402380067 | 0.714020081814801 | 0.03718854592785422 | 3.3990330978058756 | 0.5503904797322424 | 0.07437709185570844 | 1.4057270360728895 | 1.2867236891037561 | 16.444775009297143 | 0.0 | 1.2569728523614727 | 3.7486054295277054 | 1.9412420974339901 | 0.40163629602082557 | 64.16511714391969 |Rhizobiales
Acidobacteria
Actinobacteria
Figure S1. Bacterial compositions of 6 lichen samples at the phylum (A), class (B), order (C) and family (D) levels.
Fig. S1

## Slide 2
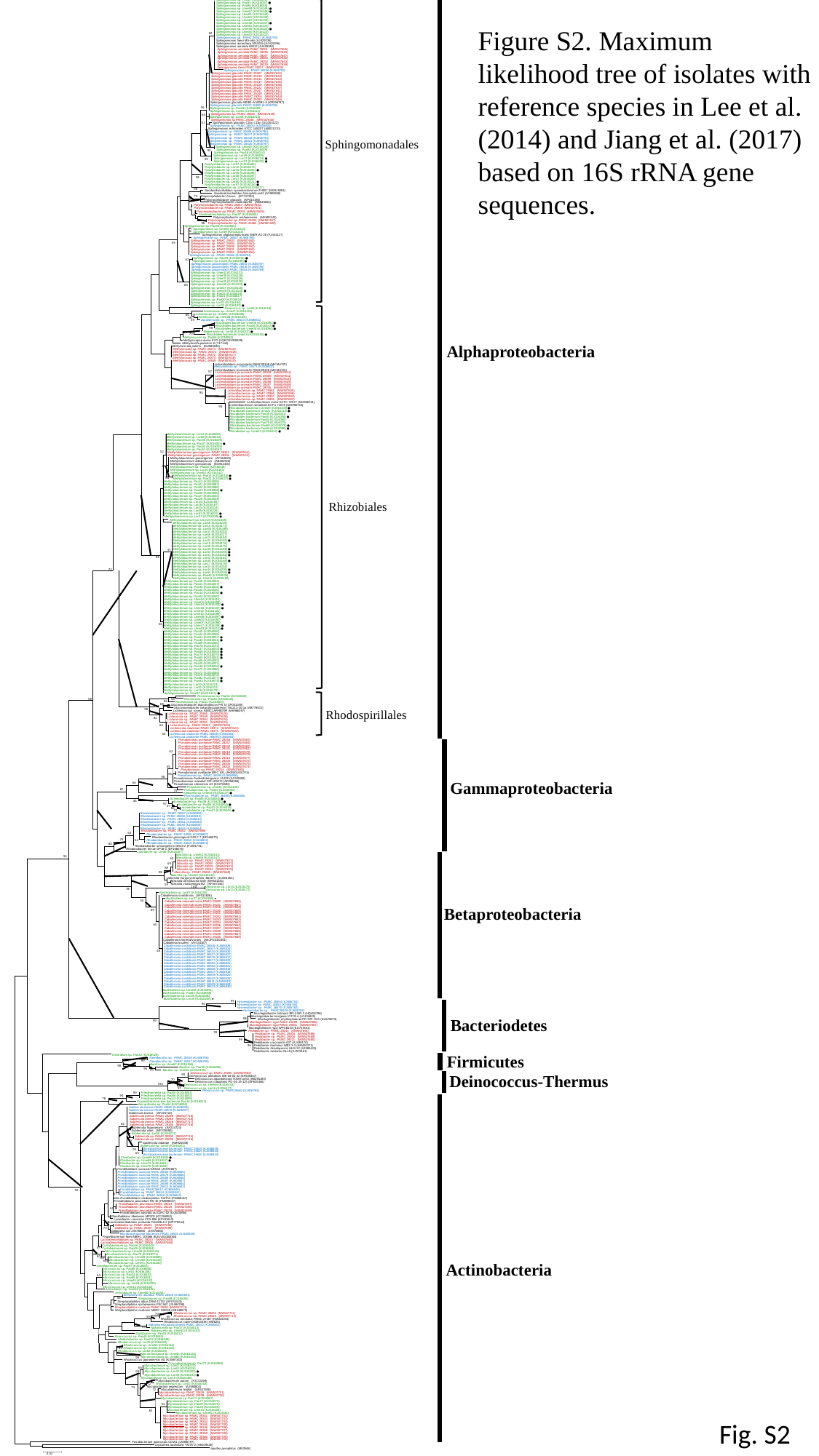

Sphingomonas sp. Pao92 (KJ016088)
 Sphingomonas sp. Pao91 (KJ016087) ●
 Sphingomonas sp. Pao90 (KJ016086)
 Sphingomonas sp. Ume59 (KJ016148) ●
 Sphingomonas sp. Ume57 (KJ016146) ●
 Sphingomonas sp. Ume51 (KJ016140)
 Sphingomonas sp. Ume50 (KJ016139)
Figure S2. Maximum likelihood tree of isolates with reference species in Lee et al. (2014) and Jiang et al. (2017) based on 16S rRNA gene sequences.
 Sphingomonas sp. Ume49 (KJ016138)
 Sphingomonas sp. Ume48 (KJ016137) ●
 Sphingomonas sp. Ume44 (KJ016133)
 Sphingomonas sp. Ume35 (KJ016124) ●
 Sphingomonas sp. Ume34 (KJ016123)
82
 Sphingomonas sp. Ume33 (KJ016122)
 Sphingomonas sp. PAMC 26561 (KJ606793)
 Sphingomonas faeni MA-olki (AJ429239)
 Sphingomonas aurantiaca MA101b (AJ429236)
 Sphingomonas aerolata NW12 (AJ429240)
 Sphingomonas aerolata PAMC 29301 (MW507655)
 Sphingomonas aerolata PAMC 29298 (MW507648)
 Sphingomonas aerolata PAMC 29297 (MW507647)
 Sphingomonas aerolata PAMC 29284 (MW507646)
 Sphingomonas aerolata PAMC 29250 (MW507643)
 Sphingomonas aerolata PAMC 29229 (MW507639)
 Sphingomonas faeni PAMC 29227 (MW507638)
 Sphingomonas sp. PAMC 26556 (KJ606792)
 Sphingomonas glacialis PAMC 29207 (MW507632)
 Sphingomonas glacialis PAMC 29210 (MW507633)
 Sphingomonas glacialis PAMC 29214 (MW507634)
 Sphingomonas glacialis PAMC 29217 (MW507635)
 Sphingomonas glacialis PAMC 29220 (MW507636)
 Sphingomonas glacialis PAMC 29222 (MW507637)
 Sphingomonas glacialis PAMC 29247 (MW507641)
 Sphingomonas glacialis PAMC 29249 (MW507642)
 Sphingomonas glacialis PAMC 29254 (MW507644)
 Sphingomonas glacialis PAMC 29283 (MW507645)
 Sphingomonas glacialis MDB1-A MDB1-A (KR258737)
 Sphingomonas glacialis PAMC 26605 (KJ606799)
56
 Sphingomonas sp. Pao86 (KJ016082)
 Sphingomonas sp. Lor50 (KJ016211)
 Sphingomonas sp. PAMC 29300 (MW507649)
64
 Sphingomonas sp. Lor54 (KJ016215)
 Sphingomonas sp PAMC 29246 (MW507640)
51
 Sphingomonas glacialis C16y C16y (GQ253122)
 Sphingomonas sp. PAMC 26530 (KJ606800)
 Sphingomonas echinoides ATCC 14820T (AB021370)
 Sphingomonas sp. PAMC 26608 (KJ606796)
Sphingomonadales
 Sphingomonas sp. PAMC 26617 (KJ606798)
 Sphingomonas sp. PAMC 26618 (KJ606794)
 Sphingomonas sp. PAMC 26621 (KJ606795)
 Sphingomonas sp. PAMC 26625 (KJ606797)
 Sphingomonas sp. Ume40 (KJ016129)
 Sphingomonas sp. Pao93 (KJ016089)
93
 Sphingomonas sp. Pao18 (KJ016014)
 Sphingomonas sp. Lor48 (KJ016209)
 Sphingomonas sp. Lor13 (KJ016174) ●
99
 Sphingomonas sp. Lor31 (KJ016192) ●
 Porphyrobacter sp. Lor07 (KJ016168)
 Porphyrobacter sp. Lor12 (KJ016173)
 Porphyrobacter sp. Lor34 (KJ016195) ●
 Porphyrobacter sp. Lor35 (KJ016196)
 Porphyrobacter sp. Lor36 (KJ016197)
98
 Porphyrobacter sp. Lor47 (KJ016208)
 Porphyrobacter sp. Lor60 (KJ016221) ●
 Porphyrobacter sp. Lor57 (KJ016218) ●
 Novosphingobium sp. Ume28 (KJ016117)
98
 Sandarakinorhabdus cyanobacteriorum TH057 (MG519281)
 Sandarakinorhabdus limnophila so42 (AY902680)
74
 Polymorphobacter fuscus (KF737330)
 Polymorphobacter glacialis (KP013180)
 Polymorphobacter multimanifer (AB649056)
 Polymorphobacter sp. PAMC 29367 (MW507630)
 Polymorphobacter sp. PAMC 29368 (MW507631)
 Polymorphobacter sp. PAMC 29335 (MW507629)
 Sandarakinorhabdus sp. Pao87 (KJ016083)
 Polymorphobacter arshaanensis (MK693143)
 Polymorphobacter sp. PAMC 29334 (MW507627)
 Polymorphobacter sp. PAMC 29362 (MW507628)
98
 Sphingomonas sp. Pao59 (KJ016055)
 Sphingomonas sp. Ume25 (KJ016114)
 Sphingomonas sp. Lor49 (KJ016210)
 Sphingomonas oligoaromativorans IMER-A1-28 (FJ434127)
 Sphingomonas sp. PAMC 26567 (KJ606790)
 Sphingomonas sp. PAMC 29303 (MW507650)
59
 Sphingomonas sp. PAMC 29304 (MW507651)
 Sphingomonas sp. PAMC 29330 (MW507652)
 Sphingomonas sp. PAMC 29331 (MW507653)
 Sphingomonas sp. PAMC 29352 (MW507654)
 Sphingomonas sp. PAMC 26560 (KJ606791)
 Sphingomonas sp. Pao19 (KJ016015) ●
62
 Sphingomonas sp. Lor25 (KJ016186) ●
 Sphingomonas paucimobilis PAMC 26502 (KJ606787)
 Sphingomonas paucimobilis PAMC 26546 (KJ606788)
 Sphingomonas paucimobilis PAMC 26548 (KJ606789)
 Sphingomonas sp. Ume42 (KJ016131)
 Sphingomonas sp. Ume39 (KJ016128)
 Sphingomonas sp. Ume37 (KJ016126)
 Sphingomonas sp. Ume31 (KJ016120)
 Sphingomonas sp. Ume30 (KJ016119) ●
85
 Sphingomonas sp. Ume27 (KJ016116)
 Sphingomonas sp. Ume29 (KJ016118) ●
 Sphingomonas sp. Pao23 (KJ016019)
 Sphingomonas sp. Pao21 (KJ016017)
 Sphingomonas sp. Pao20 (KJ016016)
 Sphingomonas sp. Lor33 (KJ016194)
 Sphingomonas sp. Lor32 (KJ016193) ●
 Paracoccus sp. Lor58 (KJ016219)
 Aureimonas sp. Ume47 (KJ016136)
 Aureimonas sp. Ume01 (KJ016090)
86
 Aureimonas sp. Ume26 (KJ016115)
66
59
 Aurantimonas sp. PAMC 26543 (KJ606801)
 Rhizobiales bacterium Ume06 (KJ016095) ●
 Rhizobiales bacterium Pao26 (KJ016022) ●
98
 Rhizobiales bacterium Ume05 (KJ016094) ●
69
 Beijerinckia sp. Lor46 (KJ016207) ●
70
 Rhizobiales bacterium Ume16 (KJ016105) ●
Alphaproteobacteria
 Methylocystis sp. Pao66 (KJ016062)
 Methylocapsa aurea KYG (JQKO01000009)
 Methylocella palustris K (Y17144)
 Methylorosula polaris (EU586035)
 Methylorosula sp. PAMC 29373 (MW507619)
 Methylorosula sp. PAMC 29371 (MW507618)
 Methylorosula sp. PAMC 29372 (MW507617)
 Methylorosula sp. PAMC 29370 (MW507616)
 Methylorosula sp. PAMC 29369 (MW507615)
 Lichenihabitans psoromatis PAMC29148 (MK344722)
 Methyloferula sp. PAMC 26571 (KJ606802)
 Lichenihabitans psoromatis PAMC29128 (MK344721)
97
 Lichenihabitans psoromatis PAMC 29309 (MW507612)
 Lichenihabitans psoromatis PAMC 29308 (MW507611)
 Lichenihabitans psoromatis PAMC 29289 (MW507610)
 Lichenihabitans psoromatis PAMC 29288 (MW507609)
 Lichenihabitans psoromatis PAMC 29287 (MW507608)
 Lichenihabitans psoromatis PAMC 29255 (MW507607)
 Lichenibacterium sp. PAMC 29361 (MW507605)
95
 Lichenibacterium sp. PAMC 29366 (MW507606)
 Lichenibacterium sp. PAMC 29357 (MW507604)
 Lichenibacterium sp. PAMC 29356 (MW507603)
 Lichenibacterium minor KCTC 72077 (MG996731)
 Lichenibacterium ramalinae KCTC 72076 (MG996730)
94
 Rhizobiales bacterium Ume20 (KJ016109) ●
 Rhizobiales bacterium Ume21 (KJ016110) ●
 Rhizobiales bacterium Pao05 (KJ016001)
 Rhizobiales bacterium Pao62 (KJ016058) ●
 Rhizobiales bacterium Pao64 (KJ016060)
 Rhizobiales bacterium Pao79 (KJ016075)
 Rhizobiales bacterium Pao80 (KJ016076) ●
 Rhizobiales bacterium Pao84 (KJ016080) ●
 Rhizobiales sp. Ume23 (KJ016112) ●
 Methylobacterium sp. Lor21 (KJ016182)
 Methylobacterium sp. Lor69 (KJ016230)
 Methylobacterium sp. Pao33 (KJ016029)
 Methylobacterium sp. Pao07 (KJ016003) ●
 Methylobacterium sp. Pao43 (KJ016039)
 Methylobacterium sp. Pao51 (KJ016047)
52
 Methylobacterium goesingense PAMC 29342 (MW507614)
 Methylobacterium goesingense PAMC 29336 (MW507613)
 Methylobacterium goesingense (AY364020)
 Methylobacterium adhaesivum (AB302928)
 Methylobacterium gossipiicola (EU912445)
60
 Methylobacterium sp. Pao50 (KJ016046)
 Methylobacterium sp. Lor20 (KJ016181)
 Sphingomonas sp. Ume53 (KJ016142)
 Methylobacterium sp. Pao14 (KJ016010) ●
84
 Methylobacterium sp. Pao31 (KJ016027) ●
 Methylobacterium sp. Pao12 (KJ016008)
 Methylobacterium sp. Pao01 (KJ015997)
 Methylobacterium sp. Pao02 (KJ015998)
 Methylobacterium sp. Pao03 (KJ015999) ●
 Methylobacterium sp. Pao09 (KJ016005)
Rhizobiales
 Methylobacterium sp. Pao27 (KJ016023)
 Methylobacterium sp. Pao28 (KJ016024)
 Methylobacterium sp. Lor24 (KJ016185)
 Methylobacterium sp. Lor26 (KJ016187)
 Methylobacterium sp. Lor53 (KJ016214)
 Methylobacterium sp. Lor65 (KJ016226)
 Methylobacterium sp. Lor61 (KJ016222) ●
 Methylobacterium sp. Lor27 (KJ016188) ●
 Methylobacterium sp. Ume19 (KJ016108)
 Methylobacterium sp. Lor55 (KJ016216)
 Methylobacterium sp. Lor10 (KJ016171)
 Methylobacterium sp. Loe38 (KJ016199)
 Methylobacterium sp. Lor41 (KJ016202)
 Methylobacterium sp. Lor66 (KJ016227)
 Methylobacterium sp. Lor23 (KJ016184)
 Methylobacterium sp. Lor22 (KJ016183) ●
 Methylobacterium sp. Lor15 (KJ016176)
 Methylobacterium sp. Lor09 (KJ016170)
 Methylobacterium sp. Lor08 (KJ016169) ●
91
 Methylobacterium sp. Lor04 (KJ016165) ●
 Methylobacterium sp. Lor01 (KJ016162) ●
85
 Methylobacterium sp. Lor03 (KJ016164)
 Methylobacterium sp. Lor05 (KJ016166) ●
 Methylobacterium sp. Lor17 (KJ016178)
 Methylobacterium sp. Lor43 (KJ016204)
 Methylobacterium sp. Lor44 (KJ016205) ●
72
 Methylobacterium sp. Lor64 (KJ016225) ●
 Methylobacterium sp. Pao40 (KJ016036)
 Methylobacterium sp. Ume11 (KJ016100)
 Methylobacterium sp. Pao36 (KJ016032)
 Methylobacterium sp. Pao41 (KJ016037)
 Methylobacterium sp. Pao35 (KJ016031) ●
 Methylobacterium sp. Pao34 (KJ016030)
 Methylobacterium sp. Pao10 (KJ016006) ●
 Methylobacterium sp. Pao04 (KJ016000)
 Methylobacterium sp. Ume24 (KJ016113)
 Methylobacterium sp. Ume04 (KJ016093)
 Methylobacterium sp. Ume14 (KJ016103) ●
 Methylobacterium sp. Ume18 (KJ016107) ●
 Methylobacterium sp. Ume12 (KJ016101)
 Methylobacterium sp. Ume10 (KJ016099)
 Methylobacterium sp. Ume08 (KJ016097) ●
 Methylobacterium sp. Ume03 (KJ016092)
 Methylobacterium sp. Ume07 (KJ016096)
56
 Methylobacterium sp. Ume17 (KJ016106) ●
 Methylobacterium sp. Ume22 (KJ016111) ●
 Methylobacterium sp. Pao42 (KJ016038)
 Methylobacterium sp. Pao44 (KJ016040)
 Methylobacterium sp. Pao61 (KJ016057) ●
 Methylobacterium sp. Pao45 (KJ016041) ●
 Methylobacterium sp. Pao69 (KJ016065)
 Methylobacterium sp. Pao78 (KJ016074)
 Methylobacterium sp. Pao37 (KJ016033) ●
 Methylobacterium sp. Pao68 (KJ016064) ●
 Methylobacterium sp. Pao74 (KJ016070) ●
 Methylobacterium sp. Pao65 (KJ016061) ●
 Methylobacterium sp. Pao06 (KJ016002)
 Methylobacterium sp. Pao25 (KJ016021)
 Methylobacterium sp. Pao38 (KJ016034) ●
 Methylobacterium sp. Pao70 (KJ016066)
 Methylobacterium sp. Pao72 (KJ016068)
 Methylobacterium sp. Pao76 (KJ016072)
 Methylobacterium sp. Pao81 (KJ016077) ●
 Methylobacterium sp. Pao83 (KJ016079) ●
 Methylobacterium sp. Lor52 (KJ016213)
 Methylobacterium sp. Lor51 (KJ016212)
 Methylobacterium sp. Lor18 (KJ016179)
 Sphingomonas sp. Ume32 (KJ016121) ●
 Roseomonas sp. Pao52 (KJ016048)
58
 Roseomonas sp. Pao48 (KJ016044)
58
86
 Roseomonas sp. Pao11 (KJ016007)
Rhodospirillales
88
 Gluconacetobacter diazotrophicus PAl 5 (CP001189)
64
 Gluconacetobacter takamatsuzukensis T61213-20-1a (AB778531)
98
 Lichenicoccus roseus KEBCLARHB70R (MK966397)
 Lichenicola sp. PAMC 29365 (MW507625)
 Lichenicola sp. PAMC 29349 (MW507626)
82
 Lichenicola sp. PAMC 29364 (MW507624)
 Lichenicola sp. PAMC 29351 (MW507623)
66
 Lichenicola sp. PAMC 29347 (MW507620)
 Lichenicola cladoniae PAMC 29374 (MW507621)
 Lichenicola cladoniae PAMC 29375 (MW507622)
53
 Lichenicola cladoniae PAMC 26568 (KJ606803)
 Lichenicola cladoniae PAMC 26569 (KJ606804)
 Pseudomonas avellanae PAMC 29256 (MW507683)
 Pseudomonas avellanae PAMC 29257 (MW507684)
 Pseudomonas avellanae PAMC 29244 (MW507682)
 Pseudomonas avellanae PAMC 29241 (MW507681)
62
 Pseudomonas avellanae PAMC 29218 (MW507679)
 Pseudomonas avellanae PAMC 29215 (MW507678)
 Pseudomonas avellanae PAMC 29213 (MW507677)
 Pseudomonas avellanae PAMC 29208 (MW507676)
 Pseudomonas avellanae PAMC 29205 (MW507675)
65
 Pseudomonas avellanae PAMC 29202 (MW507674)
 Pseudomonas sp. PAMC 29224 (MW507680)
 Pseudomonas avellanae BPIC 631 (AKBS01001374)
65
Gammaproteobacteria
 Pseudomonas sp. PAMC 26590 (KJ606806)
98
 Pseudomonas frederiksbergensis JAJ28 (AJ249382)
 Pseudomonas mandelii CIP 105273 (AF058286)
66
 Pseudomonas silesiensis A3 (KX276592)
 Pseudomonas sp. Ume41 (KJ016130)
81
 Pseudomonas sp. Pao67 (KJ016063)
75
 Klebsiella sp. Ume38 (KJ016127) ●
 Psychrobacter sp. PAMC 26498 (KJ606805)
 Acinetobacter sp. Pao88 (KJ016084) ●
46
 Acinetobacter sp. Pao39 (KJ016035) ●
97
 Acinetobacter sp. Pao58 (KJ016054) ●
67
 Acinetobacter sp. Pao22 (KJ016018)
86
 Acinetobacter sp. Pao47 (KJ016043) ●
72
 Rhodanobacter sp. PAMC 26557 (KJ606808)
 Rhodanobacter sp. PAMC 26558 (KJ606815)
 Rhodanobacter sp. PAMC 26552 (KJ606811)
 Rhodanobacter sp. PAMC 26551 (KJ606810)
 Rhodanobacter sp. PAMC 26538 (KJ606809)
 Rhodanobacter sp. PAMC 26515 (KJ606814)
 Rhodanobacter sp. PAMC 29252 (MW507685)
53
 Rhodanobacter sp. PAMC 26505 (KJ606807)
 Rhodanobacter ginsengisoli GR17-7 (EF166075)
79
 Rhodanobacter sp. PAMC 26518 (KJ606812)
64
 Rhodanobacter sp. PAMC 26519 (KJ606813)
80
 Rhodanobacter umsongensis GR24-2 (FJ821731)
 Rhodanobacter terrae GP18-1 (EF166076)
 Luteibacter sp. Lor06 (KJ016167)
 Massilia sp. Ume52 (KJ016141)
55
 Massilia sp. Ume58 (KJ016147)
65
 Massilia sp. PAMC 29242 (MW507673)
 Massilia sp. PAMC 29240 (MW507672)
69
 Massilia sp. PAMC 29219 (MW507671)
89
 Massilia sp. PAMC 29212 (MW507670)
 Massilia sp. PAMC 29206 (MW507669)
98
 Massilia sp. Ume54 (KJ016143)
 Massilia eurypsychrophila B528-3 (KJ361504)
 Massilia atriviolacea SOD (MH551481)
 Massilia violaceinigra B2 (KF267246)
59
100
 Variovorax sp. Lor14 (KJ016175)
78
 Variovorax sp. Lor11 (KJ016172)
 Burkholderia sp. Lor67 (KJ016228)
 Caballeronia sordidicola (AF512826)
 Burkholderia sp. Lor37 (KJ016198) ●
Betaproteobacteria
50
 Caballeronia mineralivorans PAMC 29209 (MW507656)
 Caballeronia mineralivorans PAMC 29211 (MW507657)
 Caballeronia mineralivorans PAMC 29216 (MW507658)
89
 Caballeronia mineralivorans PAMC 29230 (MW507659)
 Caballeronia mineralivorans PAMC 29231 (MW507660)
 Caballeronia mineralivorans PAMC 29232 (MW507661)
 Caballeronia mineralivorans PAMC 29233 (MW507662)
 Caballeronia mineralivorans PAMC 29234 (MW507663)
60
 Caballeronia mineralivorans PAMC 29236 (MW507664)
 Caballeronia mineralivorans PAMC 29237 (MW507665)
 Caballeronia mineralivorans PAMC 29238 (MW507666)
 Caballeronia mineralivorans PAMC 29239 (MW507667)
 Caballeronia mineralivorans PAMC 29245 (MW507668)
 Caballeronia mineralivorans (AEJF01000361)
 Caballeronia udeis (AY154367)
 Caballeronia sordidicola PAMC 26506 (KJ606828)
 Caballeronia sordidicola PAMC 26507 (KJ606824)
49
 Caballeronia sordidicola PAMC 26510 (KJ606826)
 Caballeronia sordidicola PAMC 26537 (KJ606827)
 Caballeronia sordidicola PAMC 26575 (KJ606817)
 Caballeronia sordidicola PAMC 26577 (KJ606823)
 Caballeronia sordidicola PAMC 26582 (KJ606822)
 Caballeronia sordidicola PAMC 26592 (KJ606821)
 Caballeronia sordidicola PAMC 26606 (KJ606818)
 Caballeronia sordidicola PAMC 26607 (KJ606816)
 Caballeronia sordidicola PAMC 26609 (KJ606820)
 Caballeronia sordidicola PAMC 26610 (KJ606825)
 Caballeronia sordidicola PAMC 26611 (KJ606819)
 Caballeronia sordidicola PAMC 26509 (KJ606829)
 Caballeronia sordidicola PAMC 26633 (KJ606830)
 Burkholderia sp. Ume02 (KJ016091)
 Burkholderia sp. Pao63 (KJ016059)
 Burkholderia sp. Lor29 (KJ016190)
 Burkholderia sp. Lor39 (KJ016200) ●
99
 Hymenobacter sp. PAMC 26554 (KJ606781)
96
 Hymenobacter sp. PAMC 26553 (KJ606780)
 Hymenobacter sp. PAMC 26570 (KJ606782)
 Hymenobacter sp. PAMC26628 (KJ606783)
Bacteriodetes
 Mucilaginibacter lutimaris BR-3 BR-3 (HQ455786)
 Mucilaginibacter terrigena 17JY9-4 (LC434629)
98
 Mucilaginibacter phyllosphaerae PP-F2F-G21 (KU878873)
99
 Mucilaginibacter rigui PAMC 29199 (MW507686)
 Mucilaginibacter rigui PAMC 29201 (MW507687)
 Mucilaginibacter rigui WPCB133 (EU747841)
 Pedobacter sp. PAMC 29243 (MW507691)
99
 Pedobacter sp. PAMC 29203 (MW507688)
 Pedobacter sp. PAMC 29204 (MW507689)
99
 Pedobacter sp. PAMC 29221 (MW507690)
 Pedobacter cryoconitis A37 (AJ438170)
96
 Pedobacter hartonius WB3.3-3 (AM491371)
Firmicutes
 Pedobacter himalayensis HHS 22 (AJ583425)
 Pedobacter lusitanus NL19 (KJ579161)
 Clostridium sp. Pao32 (KJ016028)
 Paenibacillus sp. PAMC 26516 (KJ606786)
99
 Paenibacillus sp. PAMC 26517 (KJ606785)
 Bacillus sp. Ume67 (KJ016156)
98
Deinococcus-Thermus
 Bacillus sp. Pao30 (KJ016026)
96
70
 Bacillus sp. Ume46 (KJ016135)
 Deinococcus sp. PAMC 29285 (MW507692)
90
 Deinococcus altitudinis ME-04-01-32 (EF635407)
 Deinococcus aquiradiocola TDMA-uv53 (AB265180)
70
 Deinococcus claudionis PO-04-19-125 (EF635406)
100
 Deinococcus sp. Ume64 (KJ016153)
 Deinococcus sp. Lor16 (KJ016177)
97
 Deinococcus sp. PAMC26562 (KJ606784)
 Friedmanniella sp. Pao55 (KJ016051)
99
94
 Friedmanniella sp. Pao56 (KJ016052)
 Friedmanniella sp. Pao13 (KJ016009)
78
 Propionibacteriaceae bacterium Pao16 (KJ016012)
 Nocardioides sp. Pao54 (KJ016050)
 Subtercola boreus PAMC 26580 (KJ606836)
 Subtercola boreus PAMC 26576 (KJ606837)
 Subtercola boreus (AF224722)
 Subtercola boreus PAMC 29286 (MW507719)
 Subtercola boreus PAMC 29248 (MW507718)
 Subtercola boreus PAMC 29226 (MW507717)
84
 Subtercola boreus PAMC 29198 (MW507716)
 Subtercola frigoramans (AF224723)
61
 Subtercola vilae (MF276890)
 Subtercola sp. Lor56 (KJ016217)
 Subtercola sp. PAMC 29200 (MW507714)
 Subtercola sp. PAMC 29299 (MW507715)
87
 Subtercola lobariae (KM924549)
 Subtercola sp. Lor40 (KJ016201)
 Microbacteriaceae bacterium PAMC 26615 (KJ606835)
92
 Microbacteriaceae bacterium PAMC 26629 (KJ606833)
65
 Microbacteriaceae bacterium PAMC 26630 (KJ606834)
 Clavibacter sp. Ume69 (KJ016158) ●
 Clavibacter sp. Ume68 (KJ016157) ●
 Clavibacter sp. Ume72 (KJ016161)
 Clavibacter sp. Ume70 (KJ016159)
 Frondihabitans sucicola GRS42 (JX876867)
 Frondihabitans sucicola PAMC 26565 (KJ606839)
 Frondihabitans sucicola PAMC 26579 (KJ606845)
 Frondihabitans sucicola PAMC 26586 (KJ606846)
 Frondihabitans sucicola PAMC 26587 (KJ606847)
 Frondihabitans sucicola PAMC 26588 (KJ606844)
 Frondihabitans sucicola PAMC 26613 (KJ606843)
 Frondihabitans sp. PAMC26612 (KJ606840)
90
 Frondihabitans sp. PAMC 26614 (KJ606841)
61
 Frondihabitans sp. PAMC 26616 (KJ606842)
 Frondihabitans cladoniiphilus CafT13 (FN666417)
 Frondihabitans peucedani RS-15 (FM998017)
 Frondihabitans peucedani PAMC 29223 (MW507697)
 Frondihabitans peucedani PAMC 29225 (MW507698)
65
 Frondihabitans peucedani PAMC 29228 (MW507699)
 Frondihabitans australicus E1HC-02 (DQ525859)
 Glaciihabitans tibetensis MP203 (KC256953)
 Lysinibacter cavernae CC5-806 (KP411613)
 Lacisediminihabitans profunda CHu50b-6-2 (MF770244)
 Galbitalea sp. PAMC 29281 (MW507695)
 Galbitalea sp. PAMC 29337 (MW507696)
73
 Galbitalea soli JX876866 (JX876866)
 Microbacteriaceae bacterium PAMC 26555 (KJ606838)
 Frigoribacterium faeni NBRC 103066 (BJUV01000064)
 Lacisediminihabitans sp. PAMC 29253 (MW507693)
 Lacisediminihabitans sp. PAMC 29306 (MW507694)
 Curtobacterium sp. Pao46 (KJ016042)
61
 Curtobacterium sp. Pao08 (KJ016004)
 Microbacterium sp. Ume36 (KJ016125)
 Microbacterium sp. Pao75 (KJ016071)
Actinobacteria
80
 Microbacterium sp. Ume09 (KJ016098)
63
 Microbacterium sp. Ume60 (KJ016149)
63
 Microbacterium sp. Ume71 (KJ016160)
 Amnibacterium sp. Pao57 (KJ016053)
 Micrococcus sp. Pao60 (KJ016056)
 Micrococcus sp. Lor45 (KJ016206)
 Micrococcus sp. Pao24 (KJ016020)
64
 Micrococcus sp. Pao85 (KJ016081)
 Micrococcus sp. Ume43 (KJ016132)
 Micrococcus sp. Lor59 (KJ016220)
 Micrococcus sp. Ume13 (KJ016102)
 Arthrobacter sp. Ume62 (KJ016151)
 Arthrobacter sp. Ume65 (KJ016154)
 Streptomyces anulatus PAMC 26508 (KJ606831)
56
 Streptomyces sp. Pao89 (KJ016085)
 Streptacidiphilus albus DSM 41753 (AF074415)
 Streptacidiphilus durhamensis FSCA67 (JX484798)
 Streptacidiphilus carbonis PAMC 29251 (MW507713)
 Streptacidiphilus carbonis NBRC 100919 (AB249972)
 Rhodococcus sp. PAMC 29282 (MW507711)
74
 Rhodococcus sp. PAMC 29329 (MW507712)
50
95
 Rhodococcus aerolatus PAMC 27367 (KM044053)
 Rhodococcus ruber DSM43338 (X80625)
 Nakamurella panacisegetis PAMC 26572 (KJ606832)
99
 Nakamurella sp. Pao17 (KJ016013)
 Nakamurella sp. Ume63 (KJ016152)
 Motilibacter sp. Pao15 (KJ016011)
 Kineococcus sp. Pao49 (KJ016045)
 Modestobacter sp. Pao53 (KJ016049)
 Rhodococcus sp. Lor28 (KJ016189)
 Rhodococcus sp. Ume55 (KJ016144)
86
 Rhodococcus sp. Ume56 (KJ016145)
 Rhodococcus sp. Lor68 (KJ016229)
 Micromonospora sp. Ume45 (KJ016134)
 Micromonospora sp. Ume66 (KJ016155)
99
 Rhodococcus gannanensis M1 (KX887333)
 Corynebacterium sp. Pao73 (KJ016069)
 Mycobacterium sp. Lor62 (KJ016223)
66
 Mycobacterium sp. Lor63 (KJ016224)
69
 Mycobacterium sp. Lor42 (KJ016203) ●
 Mycobacterium sp. Lor30 (KJ016191) ●
 Mycobacterium sp. Lor19 (KJ016180)
 Mycobacterium aurum (FJ172298)
 Mycobacterium sp. Lor02 (KJ016163)
72
71
 Mycobacterium neglectum (AJ580802)
73
 Mycobacterium hodleri (AF547936)
72
 Mycobacterium sp. PAMC 29328 (MW507701)
 Mycobacterium sp. PAMC 29338 (MW507700)
 Mycobacterium sp. Pao71 (KJ016067)
 Mycobacterium sp. Pao77 (KJ016073)
53
 Mycobacterium sp. Pao82 (KJ016078)
 Mycobacterium sp. Pao29 (KJ016025)
 Mycobacterium sp. Ume15 (KJ016104)
83
 Mycobacterium sp. Ume61 (KJ016150)
Fig. S2
 Mycobacterium sp. PAMC 29341 (MW507702)
 Mycobacterium sp. PAMC 29343 (MW507703)
 Mycobacterium sp. PAMC 29344 (MW507704)
 Mycobacterium sp. PAMC 29345 (MW507705)
 Mycobacterium sp. PAMC 29346 (MW507706)
 Mycobacterium sp. PAMC 29358 (MW507707)
 Mycobacterium sp. PAMC 29359 (MW507708)
 Mycobacterium sp. PAMC 29360 (MW507709)
 Mycobacterium sp. PAMC 29363 (MW507710)
 Fusobacterium gastrosuis CDW1 (LN906797)
 Leptolinea tardivitalis YMTK-2 (AB109438)
 Aquifex pyrophilus (M83548)
0.02
